# Cardiac dysfunctions in children with drug-resistant epilepsy

**DOI:** 10.3389/fneur.2024.1381293

**Published:** 2024-04-16

**Authors:** Watthana Sridech, Kamonchanok Intamul, Kwannapas Saengsin, Nattarujee Wiwattanadittakul, Rekwan Sittiwangkul, Kamornwan Katanyuwong, Suchaya Silvilairat, Chinnuwat Sanguansermsri

**Affiliations:** ^1^Department of Pediatrics, Faculty of Medicine, Chiang Mai University, Chiang Mai, Thailand; ^2^Cardiology Division, Department of Pediatrics, Faculty of Medicine, Chiang Mai University, Chiang Mai, Thailand; ^3^Neurology Division, Department of Pediatrics, Faculty of Medicine, Chiang Mai University, Chiang Mai, Thailand

**Keywords:** epilepsy, drug-resistant, cardiac dysfunctions, myocardial strain, SUDEP, biomarkers

## Abstract

**Objective:**

There were reports of cardiac dysfunction that led to sudden unexpected death in epilepsy (SUDEP) in patients with epilepsy. Early detection of cardiac dysfunction can lead to early management to prevent sudden cardiac death in these patients. The objective of our study is to assess cardiac functions in children with drug-resistant epilepsy (DRE) compared with the normal population by using a standard echocardiogram (SE), tissue Doppler imaging (TDI) and myocardial strain evaluations (MSE).

**Method:**

Twenty-seven children who have been diagnosed with DRE based on the International League against Epilepsy (ILAE) were included in the study, along with 27 children whose ages match those of the normal control group.

**Results:**

Seventeen children, median age 12 years old, were using more than four anti-seizure medications. Structural brain lesions were the most common cause of epilepsy, 55.6% (15). Generalized tonic–clonic seizures were the most common seizure type, 55.6% (15). Children with DRE had a lower early mitral valve E wave inflow velocity compared with the control group (*p* < 0.05). They also had lowered early diastolic velocities (e′) and myocardial performance index (MPI) when compared with the control group (*p* < 0.05). There was a statistically significant difference in left ventricular myocardial strain in children with DRE, with an average of −21.1 (IQR −23.5 and −19.4) and control, −25.5 (IQR −27.3 and −24.2).

**Significance:**

Children with DRE have an impairment of left ventricular diastolic function and myocardial strain, which could indicate decreased myocardial deformation and contraction compared with controls. These cardiological assessments can be used to evaluate children with DRE for early diagnosis and management of their cardiac dysfunction.

## 1 Introduction

Drug-resistant epilepsy (DRE) is defined by the inability of two well-tolerated and properly selected medications, whether used alone or in combination, to consistently achieve seizure independence despite sufficient trials (1). Twenty-five percent of children with epilepsy have received the diagnosis of drug-resistant epilepsy (DRE), putting them at an increased risk for having sudden unexpected deaths in epilepsy patients (SUDEP) (2). The incidence of SUDEP in children with DRE has been reported at approximately 1.2–1.45 per persons year (3–5). While the precise mechanism of SUDEP remains incompletely comprehended, it is predominantly believed to be a multifactorial process (6, 7). Cardiac dysfunction has been proposed as one of the leading causes of SUDEP. The reason to support this is due to postmortem findings: one-fourth of SUDEP patients have myocardial hypertrophy and fibrosis (8). Additionally, evidence of myocardial injuries in adult patients with epilepsy and subclinical left ventricular dysfunction in children with DRE were reported (9–11). The correlation between epilepsy and cardiovascular injuries was defined as the epileptic heart, which is the heart and coronary vasculature that have been injured due to chronic epilepsy. This damage occurs as a result of repeated spikes in catecholamines and hypoxemia, leading to electrical and mechanical dysfunction (12–15). Currently, numerous studies are being conducted to avoid SUDEP by employing various methods and modern technology for prompt identification and intervention.

An echocardiogram is a non-invasive procedure that is used to evaluate heart functioning and detect myocardial damage. Nevertheless, the research conducted on individuals with epilepsy, particularly in pediatric populations, was scarce. The left ventricular ejection fraction (LVEF) has been widely employed as a biomarker to assess cardiac systolic function. But numerous data points indicate that the likelihood of it being abnormal in asymptomatic patients is low (8, 16). Hence, relying just on LVEF may not provide for early detection of cardiac dysfunction, consequently exposing the patient to the risk of SUDEP. Additional echocardiography techniques, when combined with advanced multiple variables, have been investigated as indicators of myocardial dysfunction and injury. These include the measurement of mitral valve E wave inflow velocity and tissue Doppler lateral E′ wave (8, 17). Speckle-tracking echocardiography is a non-invasive technique used to assess myocardial strain. Strain measurement has been employed as an early biomarker of cardiac injury in various diseases, including epilepsy (8, 18). The strain value was different between epileptic patients and controls (8, 19). It can provide early detection of subclinical myocardial dysfunctions (8, 20). However, there is an abundance of data regarding children with DRE, and there is currently no definitive reference for strain value in pediatric populations with different age groups (21).

Our study aims to identify cardiac dysfunctions in children with DRE by utilizing a standard echocardiogram, a tissue Doppler echocardiogram (TDI), and measuring myocardial strain. This will allow us to detect any initial signs of cardiac dysfunction in patients without cardiac symptoms.

## 2 Material and method

### 2.1 Participants

The study comprised a total of 54 children and adolescents, ranging in age from 6 to 20 years. They were categorized into two groups. The epilepsy group consisted of 27 children diagnosed with DRE according to the ILAE definition were selected from the outpatient Pediatric Neurology Clinic at Chiang Mai University Hospital. The recruitment took place between August 2022 and September 2023. The study excluded children who had underlying disorders or comorbidities that could potentially cause cardiac dysfunction, including known structural heart abnormalities, a history of post-cardiac arrest or acute myocarditis and endocrine problems. The control group consisted of 27 individuals who were healthy, of similar age and gender, and visited outpatient clinics for non-medical reasons. The parents of the children enrolled in the study were provided with comprehensive information about the study and acquired a written consent from them before enrolling their children. The data was obtained from all participants, including demographic information, seizure types and epilepsy syndromes [classified by using the ILAE 2017 classification (22, 23)], seizure frequency, electroencephalography (EEG) results, number, and type of antiseizure medications. The study was approved by Chiang Mai University’s Ethics Committee.

### 2.2 Echocardiogram measurement

#### 2.2.1 Standard echocardiogram

Both patients and controls underwent an electrocardiogram (ECG) followed by a standard echocardiogram according to the study’s protocol in the outpatient Pediatric Cardiology clinic at Chiang Mai University Hospital with a standard ultrasound machine (Philips EPIQ CVx Ultrasonography). Two-dimensional echocardiography (M-mode) for parasternal long-axis view was used to measure the ejection fraction (EF) and fraction shortening (FS). An apical four-chamber view with Pulse-wave Doppler was used to evaluate early mitral inflow velocity (E), late mitral inflow velocity (A) and the E/A ratio.

#### 2.2.2 Tissue Doppler imaging echocardiogram (TDI)

An echocardiogram using Tissue Doppler Imaging (TDI) was employed to evaluate the diastolic function of the heart at the level of the mitral septal annular plane and lateral wall plane. Measurements are taken for the early diastolic velocity (e′), late diastolic velocity (a′), peak systolic velocity (S), ratio between the early mitral flow velocity (E) and early diastolic mitral velocity (e′) (E/e′), and myocardial performance index (MPI).

#### 2.2.3 Myocardial strain

**Figure 1:**
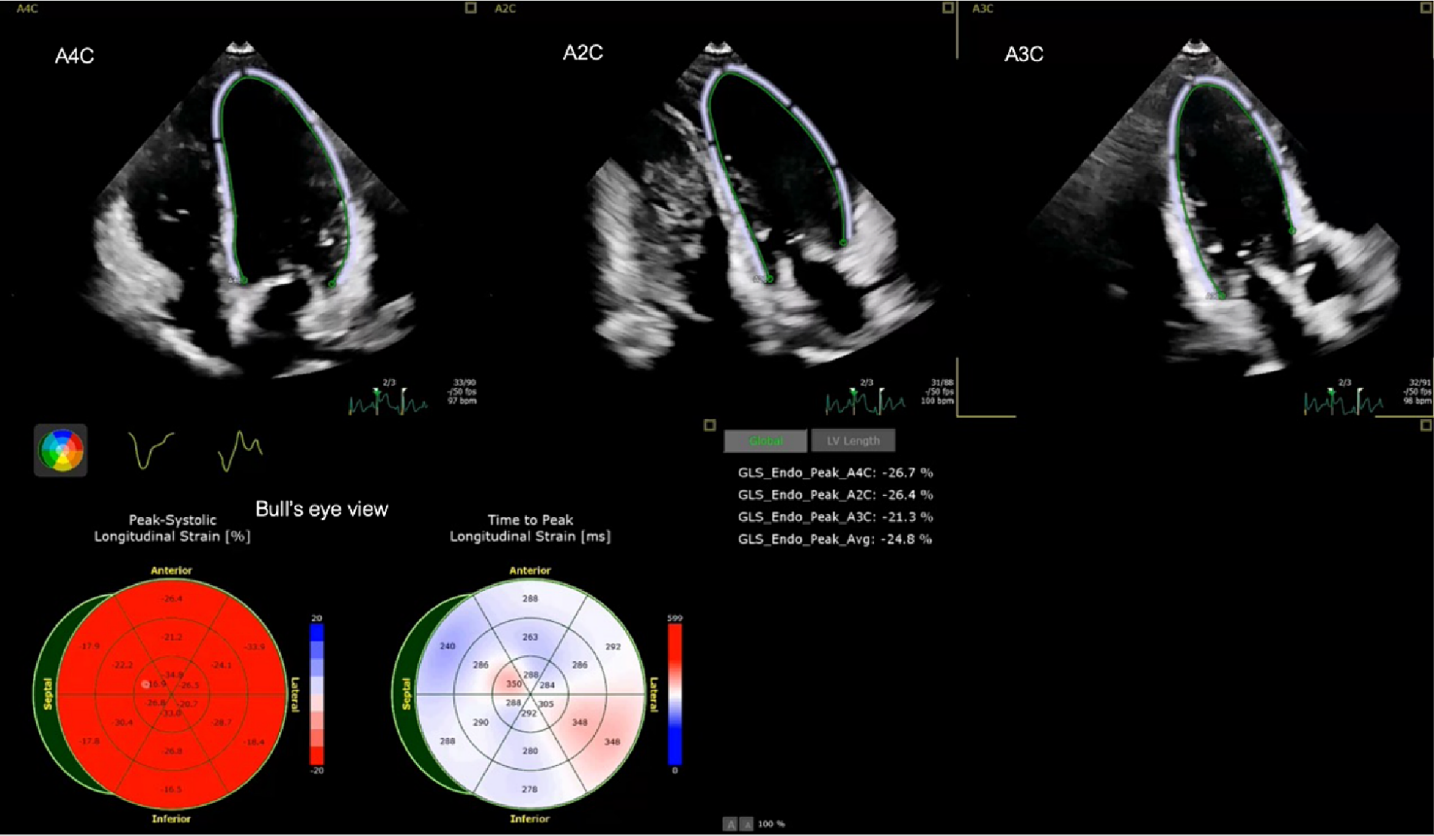
Example of left ventricular strain of a child with DRE including apical four chamber (A4C), apical two chamber (A2C), apical three chamber (A3C) and average strain (Avg) strain in Bull’s eye view.

The measurement of global longitudinal strain was performed using vendor-specific software in a semi-automated manner. The cardiologist, who was unaware of the patient’s medical history, chose the end-systolic and end-diastolic strains. The longitudinal and circumferential systolic strains are displayed as negative numbers during the contraction of the heart. The segmental strain is illustrated by three apical images: apical two chamber (A2C), apical three chamber (A3C), and apical four chamber (A4C). Additionally, a thorough visualization of the entire left ventricle is presented in a Bull’s eye view, as depicted in Figure 1.

### 2.3 Statistical analysis

The statistical analysis was performed using Stata MP version 14.1 (StataCorp LLC, Texas, United States). The categorical variables were summarized by calculating the frequency counts and percentages. The statistical tests used to calculate these values were the Chi-square test and Fisher’s exact test. The median and interquartile range (IQR) were used to summarize continuous variables. The cardiac function measurements of patients and controls were compared using an independent Mann–Whitney U test to assess the difference between the observations in the two groups. There was no additional missing data. The statistical significance was established with a *p*-value of less than 0.05.

## 3 Results

For the baseline clinical characteristics as demonstrated in Table 1, the gender and age distribution of the 27 patients and 27 controls included 16 males and 11 females, with a median age of 12 years (IQR 8–14 years). Seven children (26%) in the epilepsy group were non-ambulatory. Fourteen children (51.8%) had other medical conditions such as malnutrition (29.6%), central adrenal insufficiency (3.7%), autism spectrum disorder (11.1%) and obesity (7.4%). Only three patients (11%) were found to be currently using additional medications, namely aspirin, risperidone, and prednisolone. There was no family history of cardiac disease in any of the patients.

**Table 1:** Baseline clinical characteristic of children with DRE.

	Number (*N*)	Percentage
Gender		
Male	16	59.3
Female	11	40.7
Age		
6–10 years	11	40.7
11–15 years	11	40.7
16–20 years	5	18.6
Ambulatory status		
Walk	20	74
Non-ambulatory	7	26
Comorbid disease		
None	13	48.2
Comorbidity -Malnutrition -Central adrenal insufficiency -Autistic spectrum disorder -Obesity	14 8 1 3 2	51.8 29.6 3.7 11.1 7.4
Number of additional medications		
None	24	89
Current medication	3	11
Familial cardiac disease		
None	27	100

The seizure characteristics of the children with DRE are presented in Table 2. The main etiologies of epilepsy were structural brain lesions (15, 55.6%), genetic (5, 18.5%), and a combination of genetic and structural brain lesions (3, 11%). Focal cortical dysplasia (n = 2) was the most common in patients with structural brain lesions and Dravet syndrome with *SCN1A* mutations (*n* = 2) was the most common epilepsy syndrome in the genetic group. A majority of patients (63%) experienced seizures for a duration exceeding 10 years. At the time of the investigation, there was one patient who had been diagnosed with DRE within the past two years. For seizure types, 15 patients (55.6%) had generalized seizures and all of them were generalized tonic–clonic seizures. Seven (25.9%) had focal seizures based on their seizure semiology and EEG findings. Daily seizures were seen in 12 patients (44.4%). Seventeen patients (63%) had been using four or more current ASMs. Levetiracetam was the most common ASM used (n = 24), followed by valproic acid (n = 17) and topiramate (n = 13). None of the patients used only one ASM. Fourteen patients (51.8%) had failed at least four ASMs. There were two patients who failed only two ASMs at the time they were enrolled in the study. Twenty-five patients (92.5%) underwent to have an EEG and all of them had abnormal EEG results. Neurological imaging was performed by either computed tomography (CT) or magnetic resonance imaging (MRI) in all patients. Out of the total, 19 (70.4%) showed a structural brain abnormality.

**Table 2:** Seizure characteristics.

	Number (*N*)	Percentage
Etiology		
Genetic	5	18.5
Structure	15	55.6
Genetic and structure	3	11
Infection	1	3.7
Unknown	3	11
Duration of seizure		
Less than two years	1	3.7
Three years	1	3.7
More than five years	8	29.6
More than ten years	17	63
Seizure type		
Generalized	15	55.6
Focal	7	25.9
Focal and generalized	5	18.5
Seizure frequency		
Daily	12	44.4
Weekly	8	29.6
Monthly	5	18.5
Yearly	2	7.5
Current ASM		
Two	3	11.1
Three	7	25.9
Four	10	37.1
More than four	7	25.9
Previous ASM		
None	2	7.5
One	3	11.1
Two	3	11.1
Three	5	18.5
Four	2	7.5
More than four	12	44.3
EEG		
Not done	2	7.5
Done Abnormal	25 25	92.5 92.5
Neuroimaging		
Normal	8	29.6
Abnormal	19	70.4

Standard echocardiogram measurements for patients and controls are shown in Table 3. There were no structural cardiac abnormalities identified in both groups. All of the participants had normal sinus rhythms in their ECG results. There was a statistically significant difference in the lower left ventricular internal diameter end diastole (LVIDd) in patients in the epilepsy group compared to the control group (median 3.6 mm [IQR = 2.9–3.9] vs. 4.0 mm [IQR = 3.8–4.4], *p* = 0.001). The left ventricular internal diameter end systole (LVIDs) was lower in patients compared to controls (median 2.3 mm [IQR = 1.9–2.6] vs. 2.6 mm [IQR = 2.5–2.9], p = 0.001). The difference was statistically significant. There was no significant difference in the ejection fraction (EF) and fractional shortening (FS) of patients, which were used for cardiac systolic function assessment, compared to controls. Pulse wave standard echocardiogram revealed a significant difference in the low early mitral inflow velocity (E) (median 89.5 cm/s [IQR = 71.9–105.8] vs. 102 cm/s [IQR = 92.1–106.3], *p* = 0.027), late mitral inflow velocity (A) (median 63.4 cm/s [IQR = 51.8–71.9] vs. 54.2 cm/s [IQR = 50.5–59.1], *p* = 0.023) and E/A ratio (median 1.4 [IQR = 1.3–1.5] vs. 1.8 [IQR = 1.6–2.1], *p* < 0.001) between the patients and the control group.

**Table 3:** Standard echocardiogram.

	Patient median, (IQR)	Control median, (IQR)	*p*-value
*N*	27	27	
LVIDd(cm)	3.6 (2.9–3.9)	4.0 (3.8–4.4)	0.001
LVIDs(cm)	2.3 (1.9–2.6)	2.6 (2.5–2.9)	0.001
IVSd(cm)	0.8 (0.7–1.1)	0.84 (0.7–1.0)	0.465
IVSs(cm)	1.12 (0.98–1.26)	1.12 (0.98–1.26)	0.825
LVPWd(cm)	0.81 (0.63–1.04)	0.80 (0.71–0.93)	0.806
LVPWs(cm)	0.98 (0.77–1.13)	1.11 (0.95–1.24)	0.093
EF (%)	65.3 (61–71.3)	65.7 (60.7–68.2)	0.870
FS (%)	35.1 (32.1–39.1)	36 (32.4–38.2)	0.974
MAPSE	1.27 (1.13–1.68)	1.46 (1.23–1.71)	0.294
Pulse wave Doppler			
Early mitral inflow velocity (E) (cm/s)	89.5 (71.9–105.8)	102 (92.1–106.3)	0.027
Late mitral inflow velocity (A) (cm/s)	63.4 (51.8–71.9)	54.2 (50.5–59.1)	0.023
E/A ratio	1.4 (1.3–1.5)	1.8 (1.6–2.1)	<0.001

LVIDd, Left ventricular internal diameter end diastole; LVIDs, Left ventricular internal diameter end systole; IVSd, Interventricular septal end diastole; IVSs, Interventricular septal end systole; LVPWd, Left ventricular posterior wall end diastole; LVPWs, Left ventricular posterior wall end systole; EF, Ejection fraction; FS, Fractional shortening; MAPSE, Mitral annular plane systolic excursion.

In the TDI echocardiography (Table 4), patients exhibited a significant reduction in lateral left ventricular early diastolic velocity (e′) (median 15.4 cm/s [IQR = 12.9–17.2] vs. 18.7 cm/s [IQR = 16.7–22.0], p < 0.001) and medial ventricular early diastolic velocity (e′) (median 12.7 cm/s [IQR = 10.7–13.9] vs. 14.3 cm/s [IQR = 12.8–15.9], *p* = 0.006) compared to the control group. The myocardial performance index showed a significant elevation in patients compared with controls in both the lateral and medial walls of the left ventricles.

**Table 4:** Tissue Doppler imaging (TDI) echocardiogram.

	Patient median, (IQR)	Control median, (IQR)	*p*-value
*N*	27	27	
Lateral left ventricle			
Early diastolic velocity (e′) (cm/s)	15.4 (12.9–17.2)	18.7 (16.7–22.0)	<0.001
Late diastolic velocity (a′) (cm/s)	7.6 (6.4–9.1)	7.6 (6.1–8.9)	0.652
E′/a′ ratio	2.0 (1.7–2.3)	2.5 (2.0–3.21)	0.009
Peak systolic velocity (S) (cm/s)	9.2 (7.2–11.1)	11.0 (8.9–12.8)	0.045
Ratio between the early mitral flow velocity (E) and early diastolic mitral velocity (e′) E/e′	5.7 (4.9–7.1)	5.2 (4.7–6.1)	0.058
Myocardial performance index (MPI)	0.51 (0.43–0.63)	0.41 (0.38–0.47)	0.008
Medium left ventricle			
Early diastolic velocity (e′) (cm/s)	12.7 (10.7–13.9)	14.3 (12.8–15.9)	0.006
Late diastolic velocity (a′) (cm/s)	7.8 (6.5–8.9)	6.4 (6.0–7.5)	0.004
E′/a′ ratio	1.5 (1.3–1.8)	2.1 (1.8–2.6)	<0.001
Peak systolic velocity (S) (cm/s)	8.5 (7.4–8.8)	8.1 (7.1–8.9)	0.724
Ratio between the early mitral flow velocity (E) and early diastolic mitral velocity (e′) E/e′	7.2 (6.3–8.4)	7.0 (6.5–7.9)	0.825
Myocardial performance index (MPI)	0.50 (0.41–0.62)	0.41 (0.33–0.51)	0.011

Myocardial strains were measured for both the left ventricular (LV) and right ventricular (RV) myocardium, as shown in Table 5. The average strains in the left ventricle (LV) were substantially greater in patients compared to controls. The median strain in patients was −21.1% (IQR −23.5 to −19.4), whereas in controls it was −25.5% (IQR −27.3 to −24.2). The right ventricular free wall longitudinal strains (RVFWLS) were greater in patients than in controls, with a median of −24.5% (IQR −26.6 to −20.5%) compared to −25.5% (IQR −28.5 to −22.7%). Similarly, the right ventricular four chamber longitudinal strains (RV4CLS) were higher in patients than in controls, with a median of −20.6% (IQR −23.0 to −18.7%) compared to −23.3% (IQR −25.8 to −21.4%). Figure 1 shows an example of the myocardial strain evaluation of a child with DRE.

**Table 5:** Myocardial strain.

	Patient median, (IQR)	Control median, (IQR)	*p*- value
*N*	27	27	
Strain			
Left ventricle			
A4C	−21.2 (−23.9 to −18.48)	−25.5 (−27.3 to −24.2)	<0.001
A2C	−21.3 (−25.2 to −19.3)	−25.0 (−27.3 to −23.3)	0.005
A3C	−19.9 (−23.6 to −16.9)	−24.5 (−26.8 to −23.2)	<0.001
Average	−21.1 (−23.5 to −19.4)	−25.5 (−27.3 to −24.2)	<0.001
Right ventricle			
RVFWLS	−24.5 (−26.6 to −20.5)	−25.5(−28.5 to −22.7)	0.110
RV4CLS	−20.6 (−23.0 to −18.7)	−23.3 (−25.8 to −21.4)	0.014

A4C, apical four chamber; A2C, apical two chamber; A3C, apical three chamber; RVFWLS, right ventricular free wall longitudinal strain; RV4CLS, right ventricular four chamber longitudinal strain.

Our study attempted to evaluate the association between patient characteristics that may significantly impact cardiac function and myocardial strain. These attributes include seizure types, duration of epilepsy, frequency of seizures, and the number of antiseizure medications (ASMs). The corresponding data can be found in Supplementary Tables S1–S4. However, our subgroup analysis did not reveal significant changes in relationships.

## 4 Discussion

Our study demonstrates that children with DRE have early signs of cardiac dysfunction and myocardial injuries, specifically left ventricular diastolic dysfunctions and abnormal myocardial strains in both ventricles. This is evidenced by a significant decrease in left ventricular diastolic velocity (e′) and early mitral E wave inflow velocity and an increase in myocardial strain value in patients when compared to controls of the same age and gender. Our findings further validate the existence of the epileptic heart and enhance the comprehension of SUDEP.

Cardiac dysfunctions have been proposed and recognized as a primary factor contributing to SUDEP in both the pediatric and adult populations with epilepsy, while the supporting evidence has some limitations. Individuals diagnosed with DRE exhibited subclinical impairments in the functioning of the left ventricle of their heart (9, 11, 24). These impairments were not associated with the length of time patients had epilepsy, the type of seizures they experienced, or how often they had seizures (9). Our work provides more evidence for these findings through the utilization of both standard and TDI echocardiograms. However, our investigation did not provide evidence of the impairment of heart systolic functions, as previously recorded in the comprehensive review (25). The use of various assessment techniques for evaluating echocardiograms, the disparity in populations, and the small number of patients in each study may all have made it difficult for us to compare differences between our study and others.

Utilizing LVEF alone as an indicator of cardiac dysfunction in DRE patients may result in a delayed diagnosis since it primarily reflects cardiac systolic function. Our work, along with other reports, has demonstrated that EF does not exhibit a substantial change during the early stages of cardiac impairment (8, 9, 19). Our study proposes the utilization of pulse wave Doppler and TDI echocardiograms to evaluate the decrease in early mitral valve E wave inflow velocity, early diastolic velocity (e′) and MPI as indicators of cardiac diastolic dysfunctions in this specific group of patients. These findings were corroborated by other investigations that yielded comparable outcomes (8, 16, 17, 19).

Our investigation reveals a notable disparity in myocardial strain between the DRE group and the control group. We confirm that myocardial strain is a reliable method for non-invasively assessing early myocardial injury in children and adolescents with DRE who have normal standard echocardiogram findings. Compared to the conventional evaluation of heart systolic and diastolic functions, it is a more precise measurement for detecting myocardial injury (8, 19). According to the reference range of the Global Longitudinal Strain (GLS) result for the Philips analysis instrument, any value close to zero is considered abnormal (21, 26, 27). Nevertheless, it is important to utilize caution when interpreting the strain result, as the reference range for pediatrics and adolescents spans a large age range of 0 to 19 years old. Merely relying on the absolute value will deem the findings of diastolic function abnormalities from the Doppler and TDI echocardiograms as not clinically relevant. The disparity in strain value has the ability to elucidate the fundamental causes of myocardial injury, which may result in cardiac dysfunction in patients with DRE.

Caution should be taken when interpreting our results, as the study had a small sample size. No further support was found to suggest that anti-seizure medication has any impact on cardiovascular dysfunction. According to previous reports, adverse effects associated with the administration of anti-seizure medication on cardiac function and conduction have been documented (28–30). However, our research was unable to find a connection between the various types, quantities, and duration of anti-seizure medication and cardiac dysfunction. Furthermore, it is conceivable that cardiac dysfunction may result from several reasons, including channelopathy in genetic epilepsy syndromes such as *SCN1A* and *SCN8A*. These genetic variations have been recognized as causal factors in cardiac conduction anomalies and SUDEP (28, 31, 32). Increasing patient enrollment could improve the statistical power of the study in identifying this relationship between variables.

## 5 Conclusion

Children diagnosed with DRE exhibit compromised subclinical cardiac function, specifically in terms of left ventricular diastolic function and myocardial strain. This suggests a reduction in myocardial deformation and contraction when compared to a control group. Employing conventional echocardiography to identify noticeable structural abnormalities and reduce LVEF may result in a delay in diagnosing cardiac dysfunction in these individuals. Further research using a more extensive cohort of patients and extended follow-up assessments is necessary to evaluate the clinical result. Pulse Doppler and TDI echocardiograms in combination with MSE can be utilized to screen children with DRE for prompt identification and treatment of their cardiac dysfunction, hence mitigating the risk of SUDEP.

## Data availability statement

The raw data supporting the conclusions of this article will be made available by the authors, without undue reservation.

## Ethics statement

The studies involving humans were approved by Chiang Mai University’s Ethics Committee. The studies were conducted in accordance with the local legislation and institutional requirements. Written informed consent for participation in this study was provided by the participants’ legal guardians/next of kin. Written informed consent was obtained from the individual(s) for the publication of any potentially identifiable images or data included in this article.

## Author contributions

WS: Data curation, Formal analysis, Investigation, Writing – original draft. KI: Investigation, Writing – review & editing. KS: Data curation, Validation, Writing – review & editing. NW: Resources, Writing – review & editing. RS: Investigation, Writing – review & editing. KK: Resources, Writing – review & editing. SS: Writing – review & editing. CS: Conceptualization, Data curation, Formal analysis, Investigation, Methodology, Supervision, Validation, Writing – original draft, Writing – review & editing.
