# Supplementary material for: Cardiac dysfunctions in children with drug-resistant epilepsy
Source: Front Neurol. 2024 Apr 16;15:1381293. doi: 10.3389/fneur.2024.1381293 (PMC11058992; doi:10.3389/fneur.2024.1381293)
Supplement: Supplementary file 1 [file Table_1.docx]

Supplementary table 1. Seizure types and cardiac parameters

|  | Patient  Median, (IQR) | | P value | |
| --- | --- | --- | --- | --- |
| Seizure type | **Generalize seizure** | **Focal seizures** | **P1** | **P2** |
| N (%) | 20 (74.1) | 7 (25.9) |  |  |
| Standard echocardiogram |  |  |  |  |
| Early mitral inflow velocity(E) (cm/s) | 90.6  (73.3-105.6) | 85.7  (69.8-113.1) | 0.035 | 0.210 |
| E/A ratio | 1.4  (1.3-1.5) | 1.3 (1.3-1.6) | <0.001 | 0.010 |
| Tissue doppler imaging |  |  |  |  |
| Lateral left ventricle |  |  |  |  |
| Early diastolic velocity (e’) | 13.8  (12.3-16.8) | 17.0  (15.6-19.6) | <0.001 | 0.271 |
| E’/a’ ratio | 2.1 (1.7-2.3) | 1.9 (1.9-2.1) | 0.002 | 0.060 |
| Myocardial performance index (MPI) | 0.51  (0.42-0.61) | 0.51  (0.43-0.69) | 0.024 | 0.048 |
| Myocardial strain of left ventricle |  |  |  |  |
| Average | -20.3  (-23.9 - -18.8) | -21.9 (-23.5 - -20.4) | <0.001 | 0.014 |

Supplementary table 2. Duration of DRE and cardiac parameters

|  | Patient  Median, (IQR) | | P value | |
| --- | --- | --- | --- | --- |
| Duration of seizure | **< 10 years** | **> 10 Years** | P1 | P2 |
| N (%) | 10 (37) | 17 (63) |  |  |
| Standard echocardiogram |  |  |  |  |
| Early mitral inflow velocity(E) (cm/s) | 97.6  (85.7-111.6) | 82.7  (71.1-97.7) | 0.585 | 0.007 |
| E/A ratio | 1.4  (1.3-1.6) | 1.3 (1.2-1.5) | 0.016 | <0.001 |
| Tissue doppler imaging |  |  |  |  |
| Left lateral ventricle |  |  |  |  |
| Early diastolic velocity (e’) | 15.5  (13.6-18.4) | 15.0  (12.4-17.0) | 0.034 | 0.001 |
| E’/a’ ratio | 2.1 (2-2.3) | 1.9 (1.8-2.1) | 0.139 | 0.010 |
| Myocardial performance index (MPI) | 0.52  (0.45-0.63) | 0.46  (0.43-0.59) | 0.022 | 0.041 |
| Myocardial strain of left ventricle |  |  |  |  |
| Average | -20.3  (-23.9 - -18.8) | -21.9 (-23.5 - -20.4) | <0.001 | <0.001 |

Supplementary table 3. Seizure frequency and cardiac parameters

|  | Patient  Median, (IQR) | | P value | |
| --- | --- | --- | --- | --- |
| Frequency of seizure | Daily | Non-Daily | P1 | P2 |
| N (%) | 12 (44.4) | 15 (45.6) |  |  |
| Standard echocardiogram |  |  |  |  |
| Early mitral inflow velocity(E) (cm/s) | 82.5  (72.8-112.4) | 91.7  (72.0-105.4) | 0.086 | 0.063 |
| E/A ratio | 1.3  (1.2-1.5) | 1.5 (1.3-1.6) | <0.001 | 0.009 |
| Tissue doppler imaging |  |  |  |  |
| Left lateral ventricle |  |  |  |  |
| Early diastolic velocity (e’) | 13.1  (12.1-16.8) | 15.7  (13.7-18.4) | 0.002 | 0.015 |
| E’/a’ ratio | 1.8 (1.6-2.3) | 2.1 (1.9-2.2) | 0.007 | 0.110 |
| Myocardial performance index (MPI) | 0.50  (0.39-0.61) | 0.51  (0.44-0.69) | 0.100 | 0.011 |
| Myocardial strain of left ventricle |  |  |  |  |
| Average | -20  (-22.6 - -18.9) | -21.5 (-24.7 - -19.8) | <0.001 | <0.001 |

Supplementary table 4. Number of ASM and cardiac parameters

|  | Patient  Median, (IQR) | | P value | |
| --- | --- | --- | --- | --- |
| Number of current ASMs | **1-3** | **More than 4** | **P1** | **P2** |
| N (%) | 10 (37) | 17 (63) |  |  |
| Standard echocardiogram |  |  |  |  |
| Early mitral inflow velocity(E) (cm/s) | 82.1  (66.7-105) | 89.5  (77.6-98) | 0.110 | 0.053 |
| E/A ratio | 1.3  (1.3-1.5) | 1.4 (1.3-1.6) | <0.001 | 0.005 |
| Tissue doppler imaging |  |  |  |  |
| Left lateral ventricle |  |  |  |  |
| Early diastolic velocity (e’) | 15.4  (12.9-18.4) | 15.0  (13.6-17.0) | 0.034 | 0.002 |
| E’/a’ ratio | 1.9 (1.7-2.2) | 2.0 (1.9-2.3) | 0.016 | 0.050 |
| Myocardial performance index (MPI) | 0.52  (0.38-0.64) | 0.51  (0.45-0.59) | 0.198 | 0.006 |
| Myocardial strain of left ventricle |  |  |  |  |
| Average | -21.2  (-24.8 - -17.6) | -21.2 (-23.2 - -19.5) | 0.003 | <0.001 |
